# Cochlear Implantation in Pediatrics: The Effect of Cochlear Coverage

**DOI:** 10.3390/jpm13030562

**Published:** 2023-03-21

**Authors:** Noura Alothman, Fida Almuhawas, Reem Badghaish, Al Hanouf Alotaibi, Salman F. Alhabib, Farid Alzhrani, Abdulrahman Hagr

**Affiliations:** 1 Health Communication Sciences, College of Health and Rehabilitation Sciences, Princess Nourah bint Abdulrahman University, Riyadh 84428, Saudi Arabia; 2 King Abdullah Ear Specialist Center (KAESC), College of Medicine, King Saud University, Riyadh 11411, Saudi Arabia

**Keywords:** cochlear implantation, pediatrics, coverage, pure tone audiometry, speech perception, speech discrimination test

## Abstract

The effect of insertion depth and position of cochlear implant (CI) electrode arrays on speech perception remains unclear. This study aimed to determine the relationship between cochlear coverage and speech performance in children with prelingual hearing loss with CI. Pure tone audiometry (PTA) and speech audiometry, including speech reception threshold (SRT) using spondee words and speech discrimination score (SDS) using phonetically balanced monosyllabic words, were tested. The Categories of Auditory Performance (CAP) and Speech Intelligibility Rating (SIR) scales were also used. Thirty-one ears were implanted with the FLEX 28 electrode array, and 54 with the FORM 24 were included in the current study. For the studied ear, the mean cochlear duct length was 30.82 ± 2.24 mm; the mean cochlear coverage was 82.78 ± 7.49%. Cochlear coverage was a significant negative predictor for the mean pure tone threshold across frequecnies of 0.5, 1, 2, and 4 kHz (PTA4) (*p* = 0.019). Cochlear coverage was a significant positive predictor of SDS (*p* = 0.009). In children with cochlear coverage ≥ 82.78%, SDS was significantly better than in those with coverage < 82.78% (*p* = 0.04). Cochlear coverage was not a significant predictor of the SRT, CAP, or SIR. In conclusion, the cochlear coverage of the CI electrode array has an impact on the users’ SDS. Further long-term studies with larger sample sizes should be conducted to address the most critical factors affecting CI recipients’ outcomes.

## 1. Introduction

A cochlear implant (CI) is an electronic implantable device that is surgically placed under the skin flap over the temporal bone, with an electrode array implanted into the inner ear to replace the function of damaged hair cells in the cochlea. It is used for individuals with severe-to-profound sensorineural hearing loss without improvement using a conventional hearing aid for 3–6 months [1]. It electrically stimulates the spiral ganglion neurons and the auditory nerve, making the presence of the spiral ganglion neurons and a functional auditory nerve essential for cochlear implantation. Thus, the patency of the cochlea and the presence of the cochlear nerve in the internal auditory canal should be preoperatively determined [2].

Cochlear implantation performed on adults with severe-to-profound deafness is generally associated with improved speech perception ability [3]. However, outcomes of cochlear implantation in children have been reported to vary widely among CI users [4,5,6,7,8,9]. These variations generally depend on multiple factors such as age at implantation and onset of deafness; duration of deafness, cochlear implant experience; normality of the inner ear structure; the presence of the cochlear nerve in the internal auditory canal, residual hearing; parental participation, efficient time for daily use of the cochlear implant audio processor; and cognitive skills [4,5,6,7,8,9].

Placement of the CI electrode array inside the scala tympani (ST) compartment of the cochlea to electrically stimulate both the basal and the middle turn is another factor that may also contribute to variations in CI performance [10]. Deep insertion of an electrode array into the inner ear, theoretically, has been suggested to result in overall lower pitch perception and better spectral resolution [11,12]. However, other studies have reported that deep electrode insertion might cause pitch confusion across electrodes as a result of neural interactions between apical electrodes [13]. According to some studies, deep electrode insertion could also cause higher levels of overlapping electrical stimulation, which may result in lower speech perception [13,14].

The depth of electrode insertion depends on the surgical approach, and the type of electrode array used: whether the electrode array is pre-curved or a lateral wall electrode array. Pre-curved perimodiolar electrode arrays are designed to wrap around the modiolus of the basal turn of the cochlea. However, flexible lateral wall electrode arrays are designed to freely conform to the curvature of each cochlea, covering both the basal and middle turns of the cochlea [15].

Furthermore, the extent of cochlear coverage by a CI is influenced by several factors related to the cochlear anatomy, including the size and shape of the cochlea [16,17]. This is important because cochlear parameters can differ significantly between individuals, even in those with “normal” anatomy. For example, the length, width, and height of the cochlea can vary, affecting the overall size of the cochlear duct.

Another crucial factor in determining cochlear coverage is the length of the cochlear duct, which is determined primarily by the length and width of the basal turn of the cochlea [18]. This cochlear duct length (CDL) is defined as the length of the scala media measured from the center of the round window to the helicotrema. The basal turn is the region of the cochlea that is closest to the middle ear, and it is where the CI electrode array is inserted [16,19]. Therefore, accurate measurement of the basal turn dimensions is critical for estimating the complete length of the cochlear duct and ensuring adequate coverage of the cochlea.

Moreover, the proximity of the CI electrodes to the cochlear neurons also plays a role in determining the extent of cochlear coverage. Ideally, the electrodes should be positioned as close as possible to the neurons without causing damage to the delicate structures of the cochlea. Therefore, careful planning and positioning of the electrode array during surgery are essential for maximizing cochlear coverage and ensuring optimal hearing outcomes for patients [19,20,21,22].

Many mathematical equations and tools have been provided to compute CDL, which plays an important role in selecting the appropriate electrode array. Studies have reported that CDL could range between 25 and 45 mm. In addition, there are differences regarding gender and ethnic origins [19,20,21,22]. Therefore, the selection of electrode arrays varies in each patient [18].

Many studies have investigated the relationship between CI electrode insertion depth and patient outcomes [23]. The placement of the electrode array in the cochlea is a critical factor in determining the effectiveness of the CI, as it affects the ability of the electrodes to stimulate the auditory nerve fibers. In general, deeper insertion depths have been associated with better hearing outcomes, including improved speech perception and recognition.

However, despite significant research on this topic, the exact impact of insertion depth on patient outcomes remains unclear. Some studies have reported conflicting findings, with some showing no significant correlation between insertion depth and hearing performance. Additionally, the optimal insertion depth may vary depending on individual patient factors, such as the size and shape of the cochlea, the presence of residual hearing, and the type of electrode array used.

Another important consideration is the impact of insertion depth on pediatric cochlear implant recipients. Children’s assessment and outcome evaluation tools differ from adults, making studying the effect postoperatively more challenging. Moreover, the long-term effects of deeper insertion depths on the developing auditory system are not yet fully understood. Therefore, more studies are needed to determine the optimal insertion depth for pediatric patients and to better understand the impact of insertion depth on their hearing outcomes.

Thus, there is no agreement on a specific cochlear coverage threshold to achieve an acceptable level of speech performance, particularly in children. Therefore, this study aimed to determine the relationship between cochlear coverage and speech performance in children with prelingual hearing loss who received CI.

## 2. Materials and Methods

### 2.1. Participants

This retrospective study included children with prelingual deafness. All children who received the same device regardless of electrode type between 2014 and 2018 in a tertiary center were included in the initial enrollment. All children included had prelingual deafness; normal inner ear structure; normal development and growth; normal cochlear nerve; full-time implant use with at least 8.5 h of daily CI use; and at least two years of follow-up after implantation. The electrode array was fully inserted in these children and was confirmed by intraoperative findings and second-day radiography. The electrode array’s impedance was within the normal limit intraoperatively and postoperatively and was stable for two years after implantation.

Participants with cognitive or learning difficulties, cochlear ossifications, inner ear anomalies, abnormal cochlear nerves, or those who did not attend their regular programming and rehabilitation sessions were excluded from this study. To minimize variability due to electrode design, only the patients who were implanted with the same devices were included in the final analyses. The study was initiated after receiving ethical approval from the Institutional Review Board (Number: H-01-R-059).

### 2.2. CDL

The CDL, defined as the basilar membrane length from the round window entrance to the helicotrema, was determined in each ear implanted with a CI using temporal bone computed tomography scans interpreted by four readers with the same experience level. Then, a Cronbach’s alpha inter-rater reliability test was performed to test agreement among the four readings. The electrode length was also assessed for each individual CDL. Cochlear coverage, defined as the fraction of the cochlea covered by an electrode array, was determined using the CDL research software version 4.6 (MED-EL, Innsbruck, Austria).

### 2.3. Outcome Measures

Testing, including pure tone audiometry (PTA) and speech audiometry, was performed in a double-walled soundproof booth. Pure tone thresholds (0.25–8 kHz) were measured. The mean pure tone threshold (PTA4) was calculated across frequencies of 0.5, 1, 2, and 4 kHz. Aided speech audiometry testing in quiet, including speech reception threshold (SRT) and speech discrimination score (SDS), was also measured in the sound field through loudspeakers placed at +/−45° azimuth of participants.

The SRT was measured using spondee words, whereas SDS was tested using phonetically balanced monosyllabic words presented at 65 dB HL. The Categories of Auditory Performance (CAP) scale was used to measure speech perception performance in children with the CI. It measured supraliminal performance, which reflected everyday auditory performance in a more realistic manner. The CAP scale was defined as a hierarchical scale of auditory perceptive ability ranging between 0 (displays no awareness of environmental sounds) and 9 (uses a phone with an unknown speaker in an unpredictable context).

The Speech Intelligibility of Rating (SIR) scale was used to measure the speech intelligibility of the implanted children by quantifying their everyday spontaneous speech. This is a time-effective global outcome measure of speech intelligibility in real-life situations. SIR consisted of five performance categories ranging between “connected speech is intelligible pre-recognizable words in spoken language, the primary mode of communication may be manual” and “connected speech is intelligible to all listeners and children are easily understood in everyday context.” The speech therapist explained and clarified the CAP and SIR scales to the parents before administering them, and then the parents answered them.

### 2.4. Data Analyses

GraphPad Prism version 9.3.0 was used for all statistical analyses (GraphPad Software, La Jolla, CA, USA). The mean, standard deviation, and range (minimum and maximum values) were used to describe the characteristics of the participants. The normality of the data was first checked before comparing preoperative and postoperative data. To test the significance of the group data for normal distribution, a parametric paired t-test was used, and the Wilcoxon non-parametric test was used to test the others. A *p*-value of ≤0.05 was considered statistically significant. Correlation tests were computed to assess the relationship between cochlear coverage and the other predictors, including PTA, SRT, SDS, CAP, and SIR. Data were anonymized before analyses.

## 3. Results

Eighty-five implanted ears (57 patients) fulfilled the inclusion criteria of this study, and their data were included in the final analysis. All included patients were implanted with the same CI system (MED-EL; Innsbruck, Austria). Of these patients, 29 were unilateral CI recipients, and 28 were bilateral. Among the included ears, forty-one ears (48.2%) were implanted on the right side and 44 (51.8%) on the left side. Thirty-one ears (36.5%) were implanted with the FLEX 28 electrode array and 54 (63.5%) with the FORM 24 electrode array, as it was the available electrode in the center for a certain time. The calculated mean CDL of the included participants was 30.82 ± 2.24 mm, whereas the cochlear coverage of the inserted electrode arrays was 82.78 ± 7.49%. Table 1 summarizes the participants’ demographic characteristics, along with A-values (diameter of the basal turn), CDL, cochlear coverage, pure tone thresholds, SRT, SDS, CAP, and SIR.

**Figure 1:**
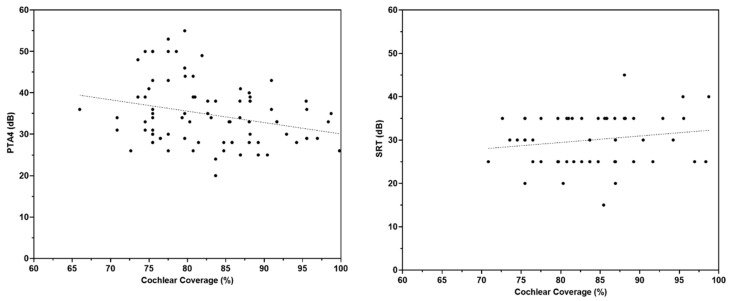
Regression analysis comparing the cochlear coverage with mean pure tone threshold (PTA4; **left panel**) and speech reception threshold (SRT; **right panel**).

The simple linear regression analysis for the X-Y plot between cochlear coverage and PTA4 (Figure 1, left panel) showed that cochlear coverage was a significant negative predictor of PTA4 (r = −0.26, *p* = 0.019). This indicated that better PTA4 was associated with greater cochlear coverage. However, cochlear coverage was not a significant predictor (r = 0.18, *p* = 0.21) of SRT (Figure 1, right panel).

The relationship between each frequency and cochlear coverage was also analyzed. The analysis revealed a statistically significant negative association between cochlear coverage and PTA at 250, 500, and 1000 Hz (Table 2). This indicated that the greater the cochlear coverage was, the better the PTA outcomes were. However, no statistical correlation was found at the higher frequencies of 2000, 4000, and 8000 Hz.

**Figure 2:**
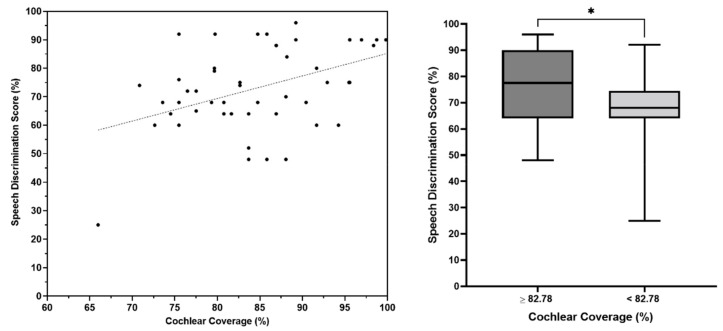
Left panel shows a regression analysis comparing cochlear coverage with speech discrimination score (SDS). The right panel shows the SDS in children with cochlear coverage ≥ 82.78% compared with children with cochlear coverage < 82.78%. * indicates statistically significant *p*-value (*p* = 0.04).

**Figure 3:**
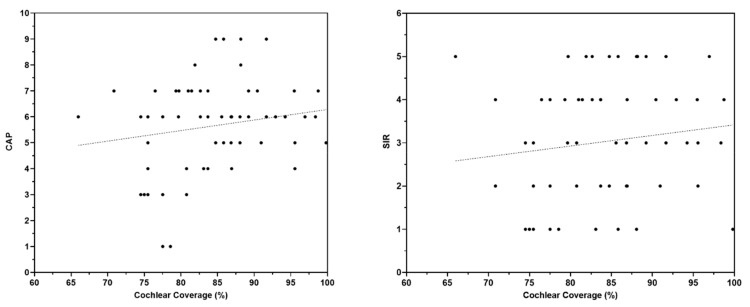
Regression analysis comparing the cochlear coverage with the Categories of Auditory Performance (CAP; **left panel**) and the Speech Intelligibility Rating (SIR; **right panel**) scales.

The analysis of the participants’ outcomes also revealed that cochlear coverage was a significant positive predictor of SDS (r = 0.37, *p* = 0.009; Figure 2, left panel). This indicated that better SDS was associated with greater cochlear coverage. Likewise, the SDS in the children with equal to or greater than 82.78% of cochlear coverage (28 patients) was statistically (*p* = 0.04; Figure 2, right panel) greater than the SDS in the children who had less than 82.78% of cochlear coverage (29 patients). Although cochlear coverage was not a significant predictor of CAP (r = 0.15, *p* = 0.23) or SIR (r = 0.18, *p* = 0.15), a positive trend was observed (Figure 3).

**Figure 4:**
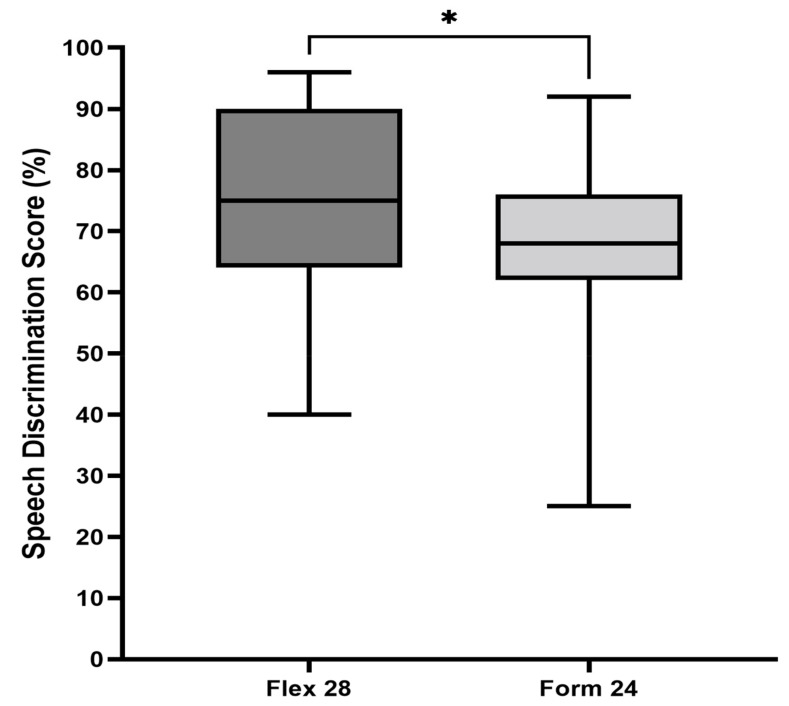
Speech discrimination scores (SDS) in children implanted with the FLEX 28 electrode array compared with children with the FORM 24 electrode array. * indicates statistically significant *p*-value (*p* = 0.031).

**Figure 5:**
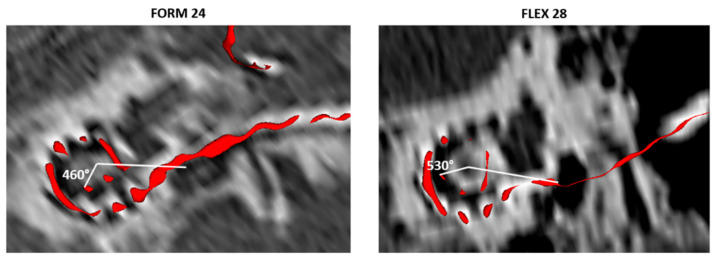
Angular depth of insertion of the FORM 24 electrode array (**left panel**) and FLEX 28 electrode array (**right panel**).

Further analyses of the individual electrodes revealed that the FLEX 28 electrode array, which had a cochlear coverage of 73.92 ± 16.06%, was associated with significantly better (*p* = 0.031) SDS compared with the FORM 24 electrode array, which had a cochlear coverage of 67.27 ± 15.12% (Figure 4). Figure 5 shows the 3D segmented angular insertion depth of the FORM 24 electrode (460°), and the FLEX 28 electrode array inserted up to 530° for illustrative purposes. These images were generated from postoperative CT scans of two patients enrolled in this study.

## 4. Discussion

The primary objective of this study was to examine the effects of cochlear coverage on the overall auditory and speech performance of prelingually deaf children with normal inner ear anatomy who underwent cochlear implantation with either the FORM 24 or the FLEX 28 electrode arrays. The results of the current study revealed that the FLEX 28 electrode array typically provided deeper coverage as compared to the FORM 24 electrode array. However, the decision to use the FORM 24 electrode array in certain patients was solely influenced by the availability of the device at the time of implantation.

Looking at the audiological outcomes, the effect of cochlear coverage on pure tone thresholds remains uncertain at this point. Based on the results of the present study, there appears to be an insignificant association between the cochlear coverage and the pure tone thresholds at high frequencies (2000, 4000, and 8000 Hz). Nevertheless, a statistically significant correlation between cochlear overage and pure tone thresholds at the other frequencies (250, 500, and 1000 Hz) was found. In general, the findings of the current study revealed that the more cochlear coverage, the better the mean pure tone threshold across 500, 1000, 2000, and 4000 Hz. This could be attributed to what has been published by Faulkner et al. [12], as the perception of low-pitched sounds could be improved by activating the entire spiral ganglion.

The results of the present investigation have shown that the cochlear coverage during cochlear implantation has a notable prognostic value for speech discrimination performance in the prelingual children who were subjects in the study. This indicates that a deeper coverage of the cochlea is positively linked to improved SDS in the investigated children.

Previous research has indicated that the best speech perception results were obtained with a cochlear coverage of 70–75% [24]. Likewise, the present study achieved a mean cochlear coverage of 82.78 ± 7.49, and the included patients showed acceptable levels of speech performance, which confirms what was previously reported. In their study, Mlynski et al. [25] reported that they achieved a mean cochlear coverage of 90.3% among their patients, with a range from 78 to 100%. However, they reported that there was no strong correlation between cochlear coverage and speech performance scores. Thus, they assumed that beyond a certain insertion depth of CI electrode arrays, no further improvement in speech perception would be expected [25]. However, the present study’s findings showed better speech discrimination performance with greater cochlear coverage. Furthermore, the current work showed that speech discrimination performance was better in the group with cochlear coverage equal to or greater than 82.78% (*p* = 0.04) compared to patients with lower coverage.

Therefore, anatomical parameters of the human cochlea should be studied prior to the CI surgery for the purpose of proper electrode array length selection. A clear understanding of the distribution and density of spiral ganglion cell bodies (SGCBs) in the cochlea, as provided in the literature review by Dhanasingh et al. [26], indicated that in both people with ‘normal’ hearing and CI recipients, SGCBs extended up to an angular depth of 680° in the cochlea. The deepest portion of the cochlea, termed segment IV, extended from 400° to 680° and showed a mean SGCB of 25% [25]. The reported SGCB distribution was consistent regardless of the type of hearing loss.

Thus, in general, placing an electrode array to match the distribution of the SGCBs in the cochlea, consequently stimulating the full range of frequencies of the cochlea, should, in theory, improve outcomes of CI. Hochmair et al. [27] have shown that stimulation of the apical region of the cochlea supported a significant degree of speech understanding and that if electrode contacts were distributed over the entire length of the cochlea, speech perception improved.

In contrast, a recent systematic review has clinically demonstrated that in six out of seven studies, there was no effect of angular insertion depth on speech perception in quiet [23]. Furthermore, only two studies of the included studies in this systematic review reported more details on the speech in noise. Both of the studies did not report a correlation between insertion depth and speech in noise scores [23]. Nonetheless, the authors concluded that the studies are flawed methodologically and that, given the considerable differences between electrode types from different manufacturers, an evidence-based conclusion on the effect of insertion depth cannot be drawn. The impact of angular insertion depth on speech performance within the first year after CI was also studied and reported. A significant positive correlation between both variables was reported in 40% of those papers [23].

It is not feasible to expect one electrode length to match all cochleae. Differences in the CDL could affect electrode placement [28], which is why electrode arrays of different lengths are available. Furthermore, the spatial difference between the electrode contacts may affect how well the electrode performs because nerve fibers from the organ of corti in the range of 632–1055 Hz are no longer radial but compressed into the apical portion of the cochlea [29], which would require optimized spacing of the electrode contacts in the perimodiolar electrode that were closer together in the apex. However, contact spacing is a complicated subject, particularly because channel interactions could occur [30,31] and be exacerbated when contacts are too closely spaced [29].

The effect of cochlear coverage on speech outcomes would also depend on the outcome measure used. The present study examined the SRT, SDS, CAP, and SIR results. Cochlear coverage was a significant predictor of SDS but not the other outcome measures. Generally, SRT measures the lowest intensity level at which a person can identify the presence of speech 50% of the time. CAP and SIR scales are based on subjective assessments [32,33], which opens up the possibility of inter-rater reliability.

A limitation of this study was that the children did not receive an appropriate language assessment test to indicate the effect of cochlear coverage on further language acquisition. Furthermore, the impact of deep insertion on apical frequency pitch confusion and residual hearing was not studied, as all the patients included in the present study had profound sensorineural hearing loss. Another limitation inherent in retrospective studies is the use of data intently collected for clinical purposes. In addition, the number of participants may have been too low to detect differences at higher purity tones. More prospective studies with multivariate regression should be performed to assess the different factors that affect CI outcomes.

## 5. Conclusions

In prelingual pediatric CI users, cochlear coverage affected speech performance in implanted children, as determined using SDS. Greater cochlear coverage ≥ 82.78% correlated with better speech discrimination performance. Before CI surgeries, a comprehensive study of the cochlear anatomy, in addition to preoperative measurements of the CDL, can help in selecting the electrode arrays that cover as much of the cochlea as possible.

## Figures and Tables

**Table 1:** Demographic characteristics of the participants. Their A-values, CDL, cochlear coverage, pure tone thresholds, SRT, SDS, CAP, and SIR, were also listed.

	Mean	Stand. Deviation	Range
Age at implantation (years)	3.23	1.46	0.8 to 5.8
A value (mm)	8.37	0.54	6.8 to 9.7
CDL (mm)	30.82	2.24	24.3 to 36.4
Cochlear coverage (%)	82.78	7.49	70 to 100
PTA4 (dB HL)	32.54	7.24	20.0 to 50.0
SRT (dB HL)	30.00	7.11	15.0 to 45.0
SDS (%)	73	27	30 to 100
CAP scale (out of 9 points)	5.55	1.83	1.0 to 9.0
SIR scale (out of 5 points)	2.98	1.42	1.0 to 5.0

Abbreviations: CAP, Categories of Auditory Performance; CDL, cochlear duct length; PTA4, mean pure tone threshold; SDS, speech discrimination score; SRT, speech reception threshold; SIR, Speech Intelligibility Rating.

**Table 2:** Results of the correlation between cochlear coverage and each frequency.

Cochlear Coverage vs.	250 Hz	500 Hz	1000 Hz	2000 Hz	4000 Hz	8000 Hz
r	−0.24	−0.25	−0.22	−0.15	−0.21	−0.22
*p* (two-tailed)	0.0290	0.0220	0.0455	0.1828	0.0542	0.0578
*p*-value summary	*	*	*	ns	ns	ns

* Statistically significant; ns: not significant.

## Data Availability

The data presented in this study are available upon request from the corresponding author. The data are not publicly available due to the ethical approval agreement.
